# The Yellow Fever Situation in New Orleans

**Published:** 1905-09

**Authors:** 


### EDITORIAL NOTES AND COMMENTS.

The Business office of THE JOURNAL-RECORD is No. 1007-1008 Century Building.

The Editorial office is 318-319-320 The Prudential.

Address all Business communications to Dr. M. B. Hutchins, Mgr.

Make remittances payable to THE ATLANTA JOURNAL-RECORD OF MEDICINE.

On matters pertaining to the Editorial and Original Communications address Dr. Bernard Wolff, Atlanta.

Reprints of original articles will be furnished at cost price. Requests for the same should always be made *on the manuscript*.

We will present, post-paid, on request, to each contributor of an original article, twenty (20) marked copies of THE JOURNAL-RECORD containing such article.

THE yellow fever situation has practically undergone no change in New Orleans. Special precautions have been taken to avoid the importation of fresh cases from the infected districts surrounding the city. The greatest difficulty appears to be in having the suspicious cases promptly reported. Upon this depends the success of the present campaign and the laws should be most rigid in regard to the matter, for not only do the neglected cases nearly always die, but they subject thousands of others to the danger of infection and perhaps death. Dr. John Guiteras, the yellow fever expert, arrived in New Orleans August 15th, and after a conference with Dr. White and an inspection of the original infected district, declared that the present methods would stamp out the fever in forty days. This seems improbable, however, from the present outlook and the serious condition at Natchez and Missisippi City.

The natural epidemic of quarantines has been the occasion of much inconvenience and in some cases unreasonable demands, and actual cruelty is alleged to have taken place.

The State Board of Health has asked the Governor to employ the National Guard in enforcing some relaxation of the most drastic quarantines in the State.

Professor Robert Boyce, of the Liverpool School of Tropical Medicine, is making a study of the methods of fighting the fever in New Orleans and will report his observations to his school. Undoubtedly many valuable points will result from the study of this disease, viewed from a standpoint entirely different from that of any former epidemic, except at Havana.

The official controversy between the physicians has been unfortunate, but *especially unfortunate* and untimely was the effort of Dr. R. B. Leach, a homeopathic physician of St. Paul, Minn., to launch his theory as to the value of arsenic in rendering the individual immune to yellow fever. There was no scientific evidence to substantiate his claims from the beginning and now that more than a dozen have died who had taken his treatment for a sufficient length of time to be immune, the most enthusiastic have given up their views as to its efficacy. Although apparently sincere in his belief, Dr. Leach was most inopportune as well as unwise to have given so much "newspaperiety" to his "would-be" remedy, of which thousands of boxes are said to have been sold.

To divert attention from real and proven methods of prevention at a crisis of this kind and cause their neglect is a very grave crime and could be most far-reaching in its effects. It is gratifying to know that owing to the numerous obstacles with which he was confronted, Dr. Leach has returned to his home in St. Paul.

---

DR. WILLIAM RININGER, a member of the faculty of Marion Sims Medical College, St. Louis, was killed August 22d by an explosion of benzine in his laboratory. He was cleaning the lens of a microscope with benzine, in the light of a gas jet, when the fumes became ignited and the explosion followed.

